# Evaluation of ex vivo produced endothelial progenitor cells for autologous transplantation in primates

**DOI:** 10.1186/s13287-018-0769-5

**Published:** 2018-01-22

**Authors:** Meng Qin, Xin Guan, Yu Zhang, Bin Shen, Fang Liu, Qingyu Zhang, Yupo Ma, Yongping Jiang

**Affiliations:** 1 Biopharmaceutical R&D Center, Chinese Academy of Medical Sciences & Peking Union Medical College, Suzhou, 215126 China; 2 Biopharmagen Corp., Suzhou, 215126 China; 3 West China Hospital, University of Sichuan, Chengdu, 610041 China; 4 School of Public Health, University at Albany, Albany, NY 12201 USA; 5 Department of Pathology, University Hospital, Stony Brook University, Stony Brook, NY 11794 USA

**Keywords:** Endothelial progenitor cells, Mobilized peripheral blood, Hepatic sinusoidal endothelium injury, Nonhuman primates

## Abstract

**Background:**

Autologous transplantation of endothelial progenitor cells (EPCs) is a promising therapeutic approach in the treatment of various vascular diseases. We previously reported a two-step culture system for scalable generation of human EPCs derived from cord blood CD34^+^ cells ex vivo. Here, we now apply this culture system to expand and differentiate human and nonhuman primate EPCs from mobilized peripheral blood (PB) CD34^+^ cells for the therapeutic potential of autologous transplantation.

**Methods:**

The human and nonhuman primate EPCs from mobilized PB CD34^+^ cells were cultured according to our previously reported system. The generated adherent cells were then characterized by the morphology, surface markers, nitric oxide (NO)/endothelial NO synthase (eNOS) levels and Dil-acetylated low-density lipoprotein (Dil-Ac-LDL) uptake/fluorescein isothiocyanate (FITC)-lectin binding actives. Furthermore, the efficacy and safety studies were performed by autologous transplantation via hepatic portal vein injection in a nonhuman primate model with acute liver sinusoidal endothelial cell injury.

**Results:**

The mobilized PB CD34^+^ cells from both human and nonhuman primate were efficiently expanded and differentiated. Over 2 × 10^8^ adherent cells were generated from 20 mL mobilized primate PB (1.51 × 10^6^ ± 3.39 × 10^5^ CD34^+^ cells) by 36-day culture and more than 80% of the produced cells were identified as EPCs/endothelial cells (ECs). In the autologous transplant model, the injected EPC/ECs from nonhuman primate PB were scattered in the intercellular spaces of hepatocytes at the hepatic tissues 14 days post-transplantation, indicating successful migration and reconstitution in the liver structure as the functional EPCs/ECs.

**Conclusions:**

We successfully applied our previous two-step culture system for the generation of primate EPCs from mobilized PB CD34^+^ cells, evaluated the phenotypes ex vivo, and transplanted autologous EPCs/ECs in a nonhuman primate model. Our study indicates that it may be possible for these ex-vivo high-efficient expanded EPCs to be used in clinical cell therapy.

## Background

Endothelial progenitor cells (EPCs) have attracted increasing attention in the fields of regenerative medicine and tissue engineering due to their excellent therapeutic potential [1, 2]. Extensive studies have demonstrated that the transplantation of EPCs into human or animals can regenerate blood vessels [3, 4], repair damaged myocardial tissue [5], treat regional ischemia [6, 7] and much more. Recently, the potential of EPCs in treating hemophilia A was claimed, attributed to the FVIII-secreting capacity of mature endothelial cells (ECs) derived from EPCs [8–10].

The origin of EPCs is interconnected with hematopoietic cells during the three waves of hematopoiesis developmental stages [11]. It has been reported that EPCs can be generated ex vivo from embryonic stem cells [12], induced pluripotent stem cells [13], and hematopoietic stem cells including those from bone marrow, umbilical cord blood, or adult peripheral blood (PB) [14, 15]. Although these cultured EPCs can incorporate into damaged vasculature and have shown notable therapeutic effects in various transplant studies [16, 17], there are still existing obstacles in terms of being ready for clinical treatment.

A major hurdle that may severely limit the transplantation of EPCs in therapeutics is the immunogenicity of endothelial cells [18, 19]. It has been reported that the endothelium expresses unique antigens, except for the common HLA antigens, and thus endothelial cells are thought to be the major target for graft rejection in HLA-identical combinations [20–22]. Therefore, the difficulty of finding a suitable match significantly restricts the availability of allogeneic EPCs in a clinical setting. For this reason, EPCs derived from autologous PB would be the most suitable resource for potential therapeutic applications. Although diverse animal disease models have been developed to evaluate the efficacy of EPCs, including murine retinal ischemia [23], rabbit carotid artery injury [24], and so forth, there are significant limitations in these small-animal models due to the remarkable species differences in terms of physiology and pathogenesis between humans and small animals [25]. Nonhuman primates are considered to be valuable preclinical models for cell transplantation studies since they are much more representative of human physiology and clinical situations than small-animal models. Thus, it is of great experimental and clinical interest to develop an EPC-transplanting model in nonhuman primates.

We previously reported a two-step culture system for the high-level ex-vivo expansion and differentiation of human EPCs derived from cord blood CD34^+^ cells [26]. Functional EPCs/ECs were efficiently generated in 3 weeks, achieving an over 1400-fold increase in proliferation. Furthermore, these EPCs/ECs were shown to reconstitute the injured sites after injection into NOD/SCID mice with acute liver sinusoidal endothelial cell (LSEC) injury. In our present research, we show that the two-step culture system is more effective by expanding and differentiating human and nonhuman primate EPCs/ECs from mobilized PB CD34^+^ cells. Subsequently, we developed a nonhuman primate LSEC injury model induced by monocrotaline (MCT), and evaluated the in vivo efficacy of generated EPCs/ECs by autologous transplantation.

## Methods

### Ethics statement

All the human cells used in this study were obtained from healthy young donors (age = 25 ± 3 years, *n* = 3) in the West China Hospital (Chengdu, China) after their written informed consent. The study was approved by the Hospital’s Ethics Committee and Research Ethics Advisory Committee (Permit Number 2014SZSLK073). All research involving animals was conducted according to relevant national and international guidelines. Male cynomolgus macaques, aged 5–7 years old and weighed 6.5–9.0 kg, were from the Medical Primate Research Center of the Institute of Medical Biology, Chinese Academy of Medical Sciences, and they were handled according to the guidelines. The experimental protocols were approved by the Yunnan Province Experimental Animal Management Association and the Experimental Animal Ethic Committee of the Institute (Permit Number SYXK-YN No. 2010-0009), following the guidelines of the US National Institutes of Health.

### Isolation of CD34^+^ cells from mobilized PB in human and nonhuman primates

The CD34^+^ cells from young healthy donors were mobilized with 5 μg/kg granulocyte-colony stimulating factor (G-CSF) for 3 days, and 200–300 mL human mobilized PB CD34^+^ cells were obtained on day 5 from each donor. Mononuclear cells (MNCs) were isolated by a Ficoll-Paque (GE Healthcare, Marlborough, MA) density gradient centrifugation. CD34^+^ cells were enriched from mononuclear cells by magnetic-activated cell sorting using a MACS Direct CD34 MicroBead Kit (Miltenyi Biotec, Amsterdam, The Netherlands) as described previously [27, 28]. Enriched CD34^+^ cells were confirmed by flow cytometry (BD Biosciences) after staining with a phycoerythrin (PE)-conjugated anti-CD34 antibody with a purity ranging from 90% to 95%.

Cynomolgus macaque PB CD34^+^ cells were mobilized with 100 μg/kg G-CSF and 50 μg/kg stem cell factor (SCF) on 5 consecutive days [27, 29]. On days 6 and 7, 15–20 mL mobilized PB were collected. CD34^+^ cells were enriched using anti-CD34 immunoglobulin (Ig)M and MACS IgM microbeads (Miltenyi Biotec) according to the manufacturer’ s instructions. Flow-cytometric analysis of purified cells was performed using an APC-conjugated anti-CD34 antibody (Beckton Dickinson, NY, USA).

### Culture of EPCs/ECs from CD34^+^ cells

The isolated CD34^+^ cells from mobilized PB of human and cynomolgus nonhuman primate were cultured by our reported two-step system [26].

Step I (0–6 days): Briefly, primate PB CD34^+^ cells were seeded in 24-well plates at a concentration of 2 × 10^5^ cells/mL in Iscove’s modified Dulbecco’s medium (IMDM) supplemented with a cytokine cocktail including SCF (200 ng/mL), Fms-related tyrosine kinase 3 ligand (Flt-3 L; 200 ng/mL), thrombopoietin (TPO; 20 ng/mL), interleukin (IL)-3 (10 ng/mL), GM-CSF (12.5 ng/mL), and vascular endothelial growth factor (VEGF; 50 ng/mL) at 37 °C in 5% CO_2_. Fresh medium with cytokines was changed on day 3.

Step II (7–36 day): The proliferated CD34^+^ cells from step 1 were further expanded and differentiated with the EBM-2 basal medium (Lonza, Switzerland) supplemented with VEGF (25 ng/mL), insulin-like growth factor (IGF; 20 ng/mL), endothelial growth factor (EGF; 10 ng/mL), basic fibroblast growth factor (b-FGF; 10 ng/mL), ascorbic acid (2 μg/mL), heparin (100 U/mL), hydrocortisone (100 ng/mL), l-glutamine (4 mM), and 20% fetal bovine serum (FBS). Approximately 1 × 10^6^ cells per well were seeded into the 24-well plates which were pre-coated with fibronectin (FN; 2.5 μg/cm^2^) at 37 °C for 2 h. The supernatant medium and suspended cells were moved out on day 9, while the adherent cells were continually cultured until day 36. The morphologic changes were observed using a microscope (Olympus Corp., Tokyo, Japan).

### Flow cytometry

The adherent cells were digested with trypsin and collected together with suspension cells in phosphate-buffered saline (PBS). Cells were then incubated with antibodies or IgG isotype control for 30 min at room temperature. The antibodies used were PE-conjugated mouse anti-human CD34 monoclonal antibody (mAb), allophycocyanin (APC)-conjugated mouse anti-human CD133 mAb, fluorescein isothiocyanate (FITC)-conjugated mouse anti-human VEGFR-2, APC-conjugated mouse anti-human CD31 mAb, FITC-conjugated mouse anti-human CD144 mAb and PE-conjugated mouse anti-human CD45mAb. Data were collected on a FACSVerse flow cytometer (Beckton Dickinson) and analyzed with FlowJo software as described previously [28, 29].

### Immunostaining

Cells were fixed with 4% paraformaldehyde for 15 min at room temperature. Primary antibodies used were rabbit anti-human CD31 (1:500; Abcam, UK) and mouse anti-human FVIII (1:500; Abcam). Cells were then incubated with fluorescent-labeled secondary anti-bodies including Cy3-labeled donkey anti-rabbit IgG (1:1000; Jackson Immuno Research, USA) and FITC-labeled donkey anti-mouse IgG (1:1000; Jackson Immuno Research). Nuclei were stained with 1 mg/mL 4,6-diamidino-2-phenylindole (DAPI; Sigma-Aldrich) for 10 min. The stained cells were imaged using a microscope (Olympus Corporation, Tokyo, Japan), and then fluorescence stained cells were counted using ImageJ software.

### Determination of nitric oxide (NO) and endothelial NO synthase (eNOS)

During the culture, the supernatants were collected and kept at –80 °C for the following nitrite detection by a NO Assay kit (Jian Cheng Bioengineering Institute, Nanjing, China). The eNOS activity in the lysis of cultured cells was tested by enzyme-linked immunosorbent assay (ELISA) according to the manufacturer’s protocol (R&D Minneapolis, MN, USA).

### Dil-ac-LDL uptake and FITC-lectin staining assay

Twenty-four hours prior to the assay, EPCs/ECs induced from nonhuman primate PB were seeded on 24-well plates in step II media without FBS for starvation treatment. Cells were then washed twice with PBS, and incubated with Dil-acetylated low-density lipoprotein (Dil-ac-LDL; 6 mg/mL; Invitrogen, Carlsbad, USA) at 37 °C in the dark for 4 h. Thereafter, cells were washed twice with PBS and stained with FITC-lectin (10 mg/mL; Sigma-Aldrich, St. Louis, USA) at 37 °C in the dark for 2 h. Subsequently, cells were fixed in cold 4% paraformaldehyde and examined with an inverted fluorescence microscope (Olympus Corporation).

### Cell labeling

The green fluorescent protein (GFP) lentivirus vector (pSSI13772) was prepared as described [10, 30]. FITC-microbeads were synthesized by conjugating microbeads with fluorescein isothiocyanate (Lumigenex Co., China; http://www.lumigenex.com). Before transplantation, the generated EPCs/ECs were double-labeled with GPF lentivirus and FITC-microbeads. First, cells were infected with GFP lentivirus for 2 h with supplemental 8 μg/mL Polybrene. Then, they were collected by trypsinization and suspended into single cells in sterile PBS followed by incubation with FITC-microbeads for 1 h at 37 °C. Labeling efficiency was approximately 100%, as confirmed by flow cytometry.

### Nonhuman primate acute liver injury model

The hepatic sinusoidal endothelium injury model in the Cynomolgus nonhuman primates was induced by the acute toxic agent MCT (Sigma-Aldrich, St. Louis, MO, USA) at a dose of 100 mg/kg through intraperitoneal injection 24 h prior to the cell transplantation. This model has been validated in the evaluation of ex vivo-produced endothelial cells in previous studies [26, 31].

### Autologous transplantation of EPCs/ECs into nonhuman primate models

Ten primates with acute liver injury were randomly divided into three groups: control group (*n* = 3), post-transplantation 7 days (*n* = 3) and post-transplantation 14 days (*n* = 4). They were all anesthetized with an intraperitoneal injection of FFM mix (2.5 mg fluanisone, 0.105 mg fentanylcitrate, and 1.25 mg midozalam HCl/kg in H_2_O). Each primate was autologously transplanted with 2 × 10^8^ EPCs/ECs (500 μL in saline) via hepatic portal vein injection using 27-gauge needles (Hamilton 90131, Switzerland) [10]. On days 7 and 14 post-transplantation, the primates were euthanized and the livers were removed following heart perfusion with ice cold saline and fixed in 4% paraformaldehyde for further detection.

### Detection of transplanted EPCs/ECs

The hepatic tissues were embedded in optimum cutting temperature (OCT; Sakura, Tokyo, Japan) compound to make the cryosections (20 μm; Leika CM1900, Germany) and the GFP lentivirus infected EPCs/ECs were analyzed by the immunofluorescence method. A rabbit anti-GFP primary antibody (1:100, Santa Cruz) and a Cy3-labeled donkey anti-rabbit IgG secondary antibody (1:1000, Jackson Immuno Research) were used. Nuclei were counterstained with 1 mg/mL DAPI (Sigma) for 10 min. These cryosections were then observed under a fluorescence microscope (Olympus Corporation). The GFP- and FITC-microbead-labeled cells and the total cells in each section were quantified using ImageJ software, and the average percentages of the labeled cells in the total cells were calculated from 25 sections per monkey.

### Statistical analysis

Results are expressed as mean ± SD. For each group, at least three independent experiments were analyzed. All data were subjected to analysis of variance (ANOVA) followed by Fisher’s analysis for comparison between two means. Differences are considered as significant when *P* value < 0.01.

## Results

### Expansion and differentiation of human EPCs derived from mobilized PB CD34^+^ cells

**Fig. 1:**
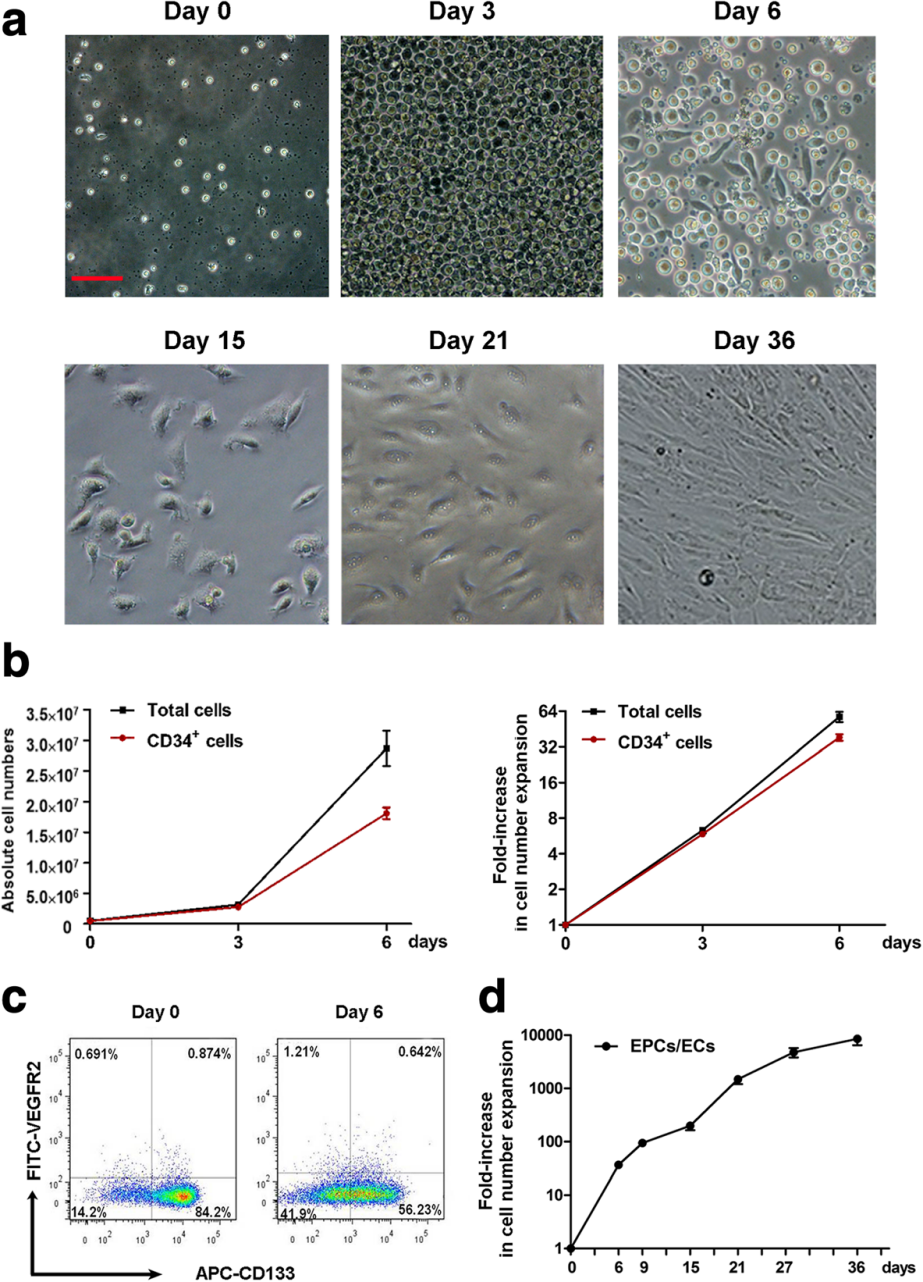
The expansion and differentiation of EPCs derived from CD34^+^ cells of human PB. The isolated human PB CD34^+^ cells were cultured in modified IMDM medium supplemented with human cytokine combinations for the first 6 days. Then, the adhering endothelial progenitor cells (EPCs)/endothelial cells (ECs) were subsequently differentiated in EBM-2 basal medium with endothelial growth factors from 7 days; the cell numbers and expansion folds were calculated at different time points. **a** Cell morphology imaged with an optical microscope on days 0, 3, 6, 15, 21, and 36 (scale bar = 50 μm). **b** (left) Absolute number of total cells and CD34^+^ cells from day 0 to day 6; (right) fold-increase in cell number expansion of total cells and CD34^+^ cells from day 0 to day 6. **c** The expression of CD133 and VEGFR2 in the early EPCs from day 0 to day 6. **d** Expansion fold of human EPCs/ECs over the initial EPCs derived from human PB CD34^+^ cells from day 0 to day36. The data represent means ± SD, *n* = 3

Previously, we had efficiently generated human EPCs/ECs from cord blood CD34^+^ cells with a remarkable improvement in the yield by a two-step culture system. We here applied this culture technology to generate EPCs/ECs from human mobilized PB CD34^+^ cells as source of autologous EPCs. Firstly, mobilized PB CD34^+^ cells were cultured in the step I medium for abundant expansion of CD34^+^ cells and early EPCs. The initial percentages of CD34^+^ and CD133^+^/VEGFR2^+^ cells were 94.6 ± 1.25% and 0.87 ± 0.09%, respectively. Within 6 days cells exhibited robust suspension growth, and a proportion of cells had started to adhere onto the plates indicating the characteristics of early EPCs (Fig. 1a, day 6). The total cell number increased from 5 × 10^5^ to 2.92 × 10^7^ ± 2.44 × 10^6^, showing a ~60-fold proliferation (Fig. 1b). The percentages of CD34^+^ cells were maintained at a relatively high level of 63.3 ± 2.93% and the expression of CD133/VEGFR2 marker was still low at 0.63 ± 0.17% (Fig. 1c). Subsequently, the expanded cells were transferred to the step II medium for further adherent induction and differentiation toward EPCs. Three days later (day 9), a number of increasing cells began to exhibit adherent phenotypes but with irregular cell morphology. Afterwards, the suspended cells were completely removed, and adherent cells were continuously cultured in the same medium. From day 15 to day 36, almost all cells showed a typical spindle-like shape and they arrayed uniformly like pitching stones in culture (Fig. 1a, days 15, 21, and 36). On day 21, the absolute number of EPCs reached 6.45 × 10^6^ ± 3.05 × 10^5^, about a 1500-fold expansion compared with the cell number on day 0. After further culture, the EPC number reached 3.70 × 10^7^ ± 2.76 × 10^6^ on day 36, ultimately achieving an 8534.75 ± 532.83-fold increase (Fig. 1d). Collectively, these results demonstrated that the two-step culture system was efficient for the ex-vivo expansion and differentiation of EPCs/ECs derived from human mobilized PB CD34^+^ cells.

### Characterization of EPCs/ECs derived from human PB CD34^+^ cells

**Fig. 2:**
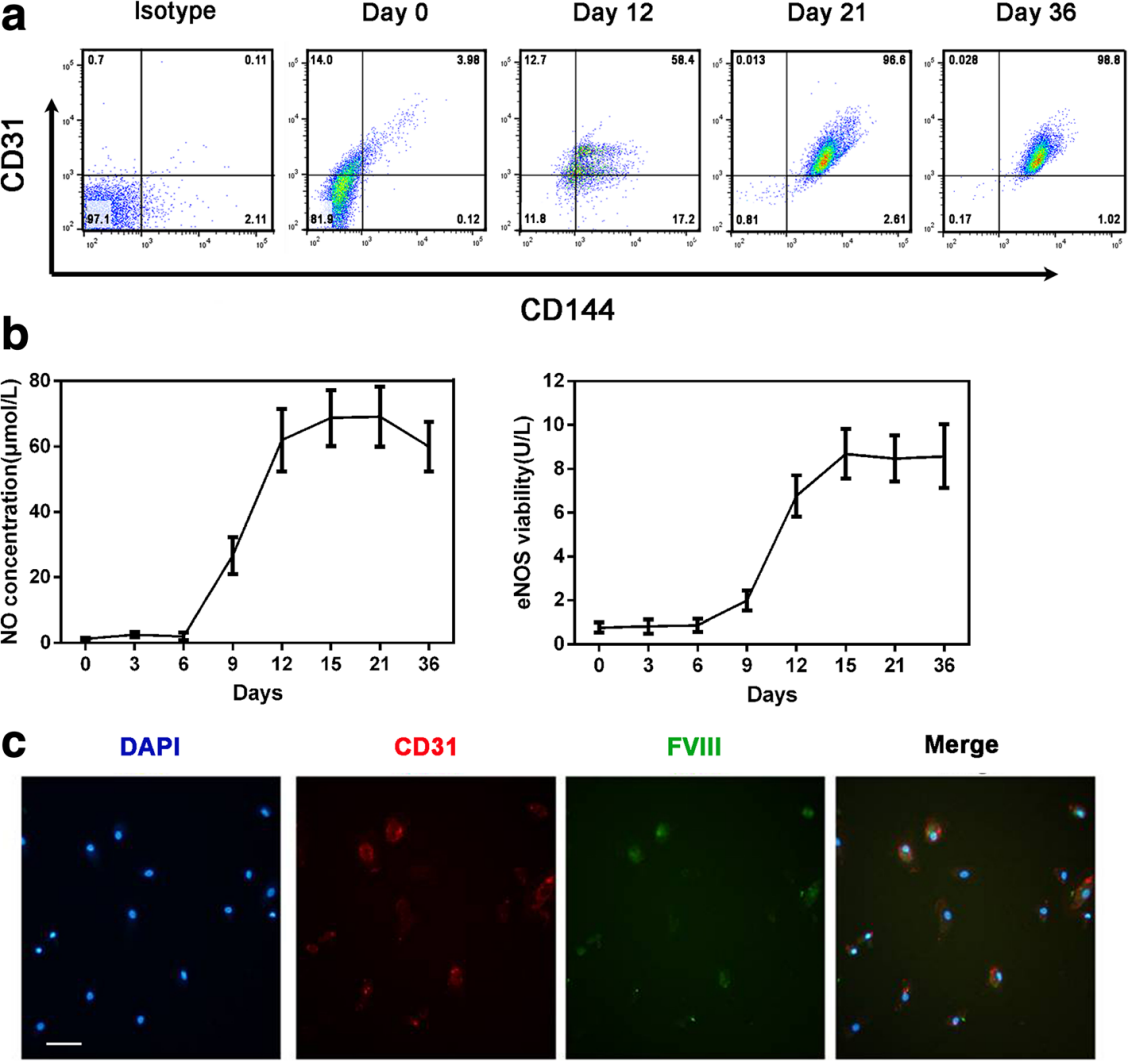
Phenotypic and functional analysis of EPCs produced from mobilized human PB CD34^+^ cells. The characterization of the generated EPCs/ECs was analyzed by mature endothelial cell markers CD31 and CD144, NO production measurement, and CD31 and FVIII double staining. **a** Representative flow cytometry profiles of CD31 and CD144 cell markers on days 0, 12, 21, and 36. **b** Measurement of nitric oxide (NO) concentration and endothelial NO synthase (eNOS) viability during the culture process. **c** Immunofluorescence analysis of CD31 on cell surface (red) and FVIII in cytoplasm (green) on day 21 (scale bar = 20 μm)

The cell surface markers during the cell culture were analyzed on days 0, 12, 21, and 36 by flow cytometry. During the differentiation, the expression levels of the EC-specific markers CD31^+^ and CD144^+^ increased continuously, with the frequency of CD31^+^/CD144^+^ (late stage expression of EPCs/ECs) at 96.6% ± 1.4% by day 21 and sustained at 98.8 ± 2.4%until day 36 (Fig. 2a). Furthermore, the levels of eNOS expression and NO release were also determined during the culture process. A high NO concentration in the culture supernatant was detected from day 9 and become stable after day 15. eNOS in the cultured cells was detectable from day 9, after which a stable growth was observed from day 12 to day 21 (Fig. 2b). These results were consistent with the double-stain by red and green fluorescence in which over 90% adherent cells on day 21 exhibited positive expression for CD31 and FVIII (Fig. 2c).

### Generation of EPCs/ECs derived from nonhuman primate mobilized PB CD34^+^ cells

**Fig. 3:**
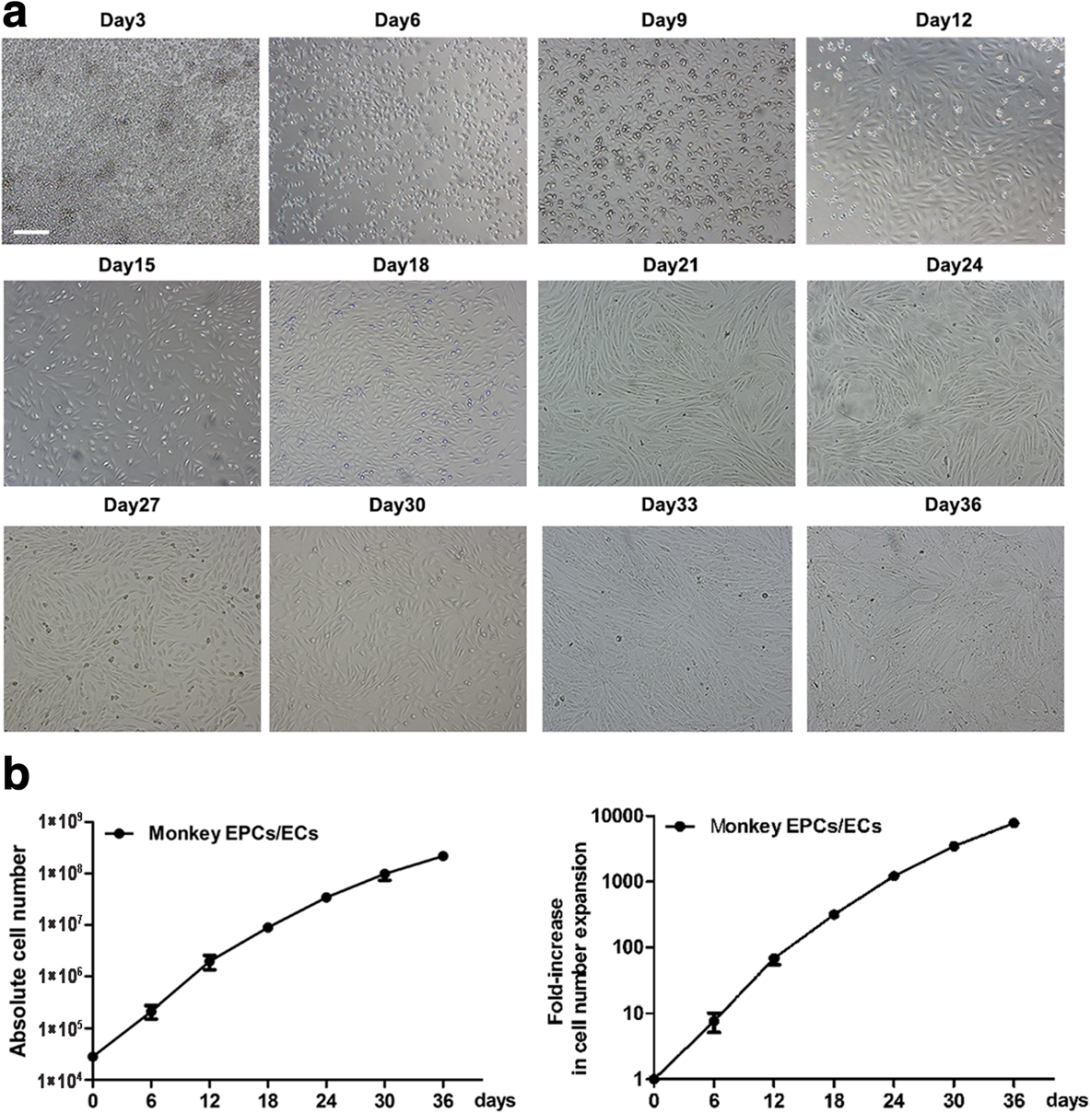
The expansion and differentiation of EPCs derived from CD34^+^ cells of nonhuman primate PB in vitro. The isolated nonhuman primate PB CD34^+^ cells were expanded and differentiated into endothelial progenitor cells (EPCs)/endothelial cells (ECs). **a** The morphology of induced EPCs was photographed by microscope every 3 days from day 0 to day 36 (scale bar = 50 μm). **b** Absolute cell number and fold-increase of EPCs/ECs over the initial CD34^+^/VEGFR2^+^ EPCs isolated from nonhuman primate PB. Data represent mean ± SD; *n* = 10

Nonhuman primate mobilized PB samples (15–20 mL) were collected after a G-CSF/SCF conditioning regimen, and 1.51 × 10^6^ ± 3.39 × 10^5^ CD34^+^ cells were isolated with a purity of 84.6 ± 4.3%. After a 6-day suspension culture followed by another 30-day adherent culture, morphological observation during the culture period indicated that primate EPCs were similar to human EPCs in terms of the shape, size, and arrangement (Fig. 3a). The number of generated nonhuman primate EPCs reached 2.18 × 10^8^ ± 1.80 × 10^7^, equivalent to a 7766 ± 796.6-fold increase compared to the initial CD34^+^/VEGFR^+^ EPCs (Fig. 3b).

### Characterization of generated nonhuman primate EPCs/ECs

**Fig. 4:**
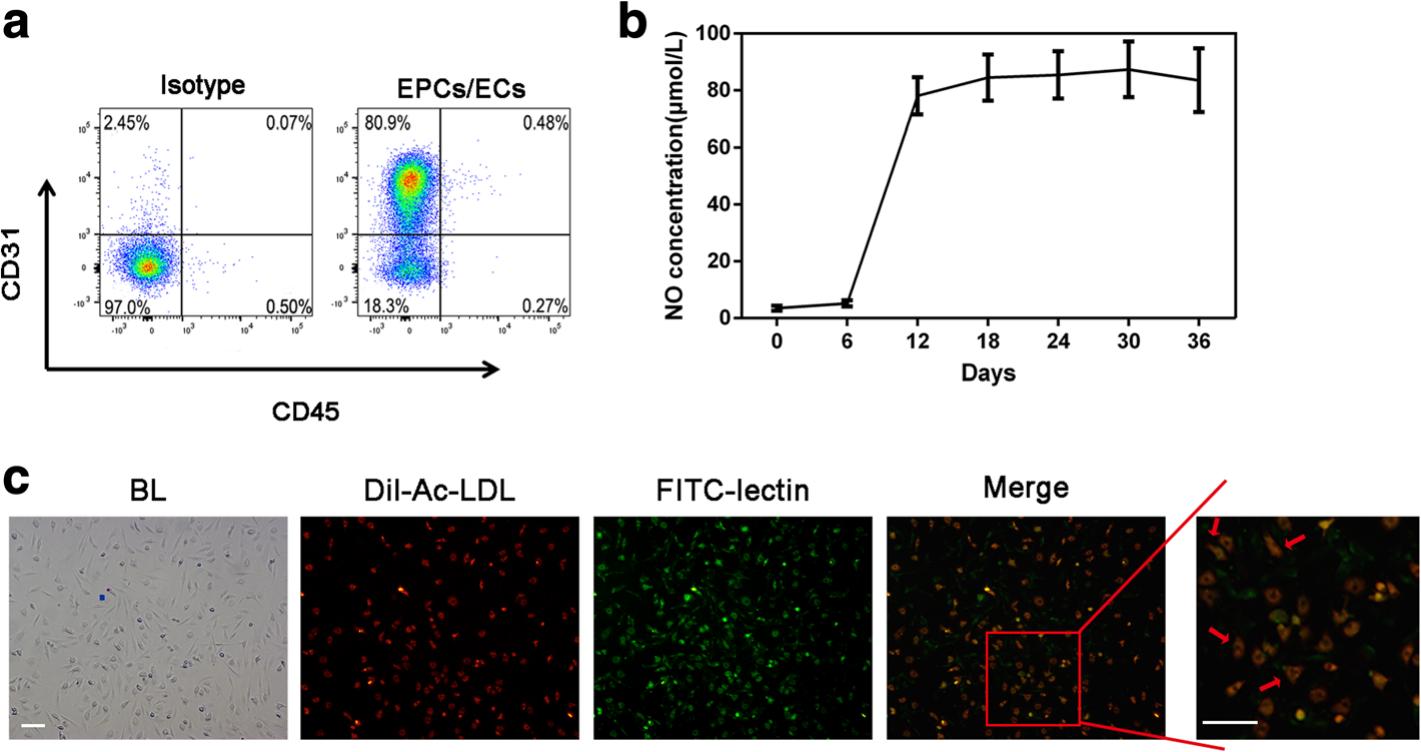
Characterization of produced EPCs/ECs derived from nonhuman primate PB CD34^+^ cells. **a** Representative flow cytometry profiles of CD31 and CD45 cell markers of produced nonhuman primate endothelial progenitor cells (EPCs)/endothelial cells (ECs) on day 36. **b** The concentration of nitric oxide (NO) released by produced nonhuman primate EPCs/ECs during the culture. **c** The purities and phenotypes of induced EPCs/ECs were assessed by Dil-acetylated-low density lipoprotein (Dil-Ac-LDL) and fluorescein isothiocyanate (FITC)-lectin double staining for nonhuman primate cells. The florescent densities were scanned by microscope and the double staining cells were identified as functional EPCs/ECs

The generated nonhuman primate EPCs/ECs were identified as CD31^+^/CD45^–^ cells. Figure 4a shows that the endothelia marker CD31 was highly expressed and that no hematopoietic marker CD45 was detected. The NO release started from day 6 and became stable from day 12 (Fig. 4b). In addition, the primate EPCs were characterized by FITC-lectin staining and Dil-Ac-LDL uptake on day 36. Figure 4c demonstrates that nearly all the adherent cells were bound with FITC-lectin and that over 80% of them were capable of taking in Dil-ac-LDL, indicating a normal function of endothelial cells.

### Autologous transplantation of EPCs in a nonhuman primate model

**Fig. 5:**
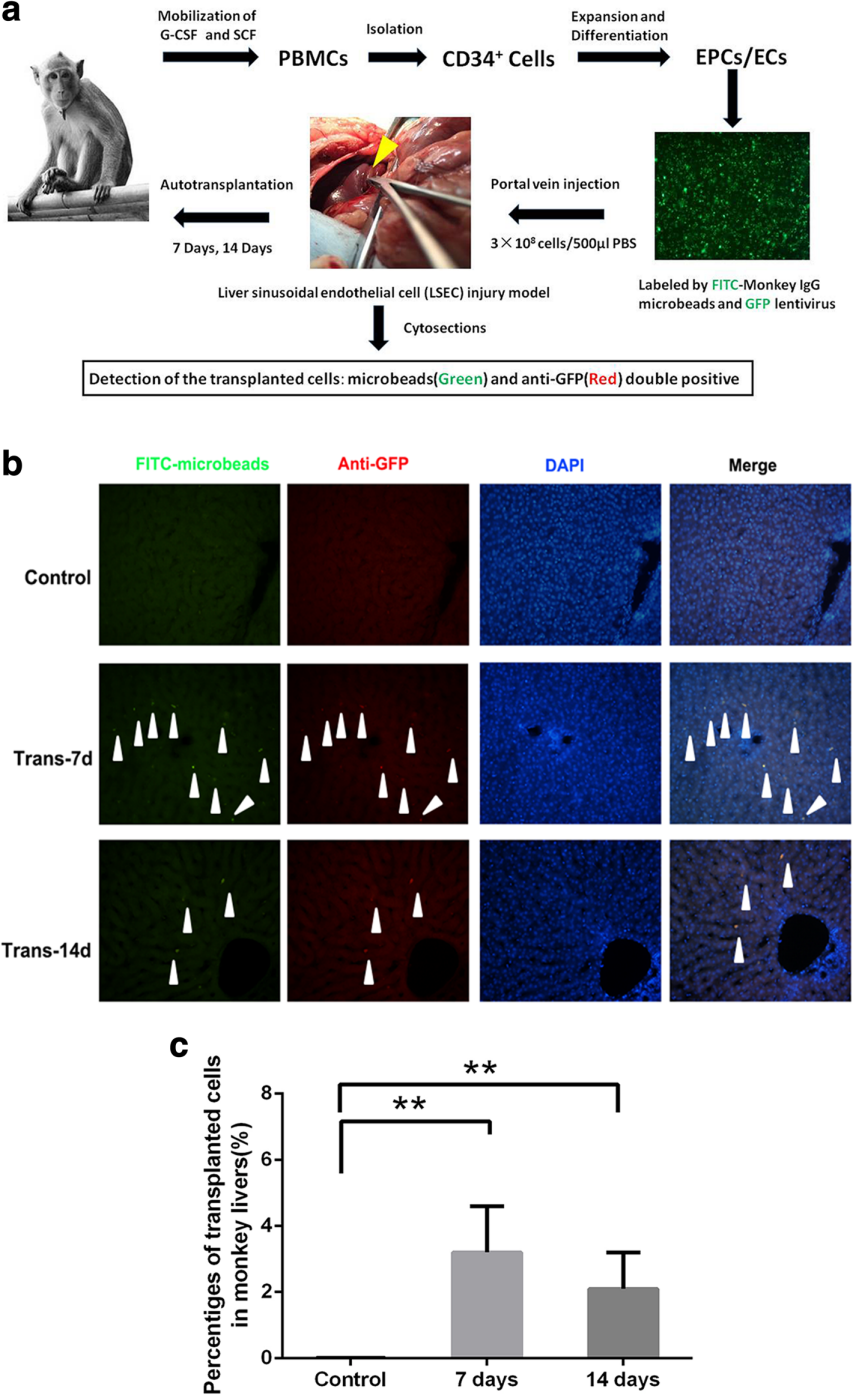
Autologous transplantation of induced EPCs/ECs into the livers of cynomolgus nonhuman primates. The cultured and labeled nonhuman primate EPCs/ECs were transplanted to livers with sinusoidal endothelium injury at a dose of 3 × 10^8^/500 μL saline in each nonhuman primate through hepatic portal vein injection. The hepatic tissues were sectioned on day 7 (Trans-7d; *n* = 3) and day 14 (Trans-14d; *n* = 4) post-transplantation, as well as from the control group (*n* = 3). **a** The technical method of autologous transplantation in nonhuman primates. **b** The GFP- and FITC-microbead-labeled cells were detected in cross-sections of nonhuman primate livers; scale bar indicates 50 μm. **c** The percentages of transplanted cells were calculated (25 sections per nonhuman primate). The data represent means ± SD; **P < 0.01 compared with the control group. EC endothelial cell, EPC endothelial progenitor cell, FITC fluorescein isothiocyanate, G-CSF granulocyte-colony stimulating factor, GFP green fluorescent protein, PBMC peripheral blood mononuclear cell, PBS phosphate-buffered saline, SCF stem cell factor

To assess the safety and efficacy of ex vivo-generated EPCs for clinical application, autologous transplantation of EPCs was employed in a nonhuman primate model. The trial design is outlined in Fig. 5a. The primate LSEC injury model was induced by 100 mg/kg MCT, which is a toxic agent that can disrupt the sinusoidal endothelial barrier and stimulate the incorporation of transplanted cells into liver parenchyma. Primates in the post-transplantation 7 day (*n* = 3) and 14 day (*n* = 4) groups were injected with autologous EPCs (2 × 10^8^ cells/500 μL in saline) double-labeled with GFP and FITC-microbeads, whereas the control group primates (*n* = 3) were treated with 500 μL saline though the hepatic portal vein (Fig. 5b). Four to six hours after transplantation, all nonhuman primates had recovered from the portal vein injection and gradually resumed normal diets and behaviors. Primate routine blood analysis and liver functional enzymes were at the normal level, and no apparent side effects were observed. About 3.2 ± 1.4% and 2.1 ± 1.1% of liver cells were observed as Dil-ac-LDL and FITC-lectin double positive in the liver cryosections (25 sections per nonhuman primate) on days 7 and 14, respectively, indicating that transplanted EPCs were capable of repopulating into functional ECs in vivo (Fig. 5c). Furthermore, the injected EPCs/ECs were scattered in the intercellular spaces of hepatocytes at the hepatic tissues on day 14 (Fig. 5b), suggesting that the transplanted cells could migrate towards injured LSEC sites in nonhuman primate livers.

## Discussion

Autologous transplantation of EPCs does not involve pre-transplant crossmatch typing or post-transplant immunological rejection, indicating a promising treatment method for various diseases such as myocardial ischemia and hemophilia A in the clinic. Because of the poor cell number of directly isolated EPCs from peripheral blood, ex vivo expansion and differentiation of EPCs is particularly important. We previously reported a two-step culture system for the large-scale generation of human EPCs derived from CD34^+^ cells from human cord blood ex vivo [26]. However, considering the therapeutic purpose, CD34^+^ cells from human mobilized PB are safe and more practically convenient for autologous transplantation in humans. In this study, the final numbers of proliferated and differentiated EPCs from mobilized PB were higher than from human cord blood (Fig. 1d), due to more CD34^+^ cells being isolated from the mobilized PB compared with the cord blood. Notably, over 2 × 10^8^ EPCs/ECs were generated from 15–20 mL mobilized PB (about 1.51 × 10^6^ ± 3.39 × 10^5^ CD34^+^ cells), up to 350-fold higher than that reported previously [16, 32, 33]. Nonhuman primates are considered as the optimal animal model for evaluating the safety and function of cell preparations for preclinical studies. Unfortunately, strong auto-immunity rejection would be induced with the direct use of human EPCs in nonhuman primates; thus, we applied our culture system to generate EPCs derived from nonhuman primate mobilized PB CD34^+^ cells for evaluation of preclinical studies and potential clinical application in the future.

The endothelial cells derived from rhesus monkeys share a cell surface phenotype similar to humans and reportedly can be cultured ex vivo [34]. Our novel culture system technique from previous protocols isolated, purified, and cultured EPCs from normal PB mononuclear cells. We chose mobilized PB and enriched CD34^+^ cells as the initial component for expansion and induction of EPCs, as well as using nonhuman primate PB CD34^+^ cells. It has been reported that EPCs and hematopoietic stem cells originate from a common precursor, the hemangioblast, and share a number of cell-surface markers [35]. Eunju et al. suggested that culture of the CD34 fraction is more efficient for EPC expansion than that of mononuclear cells [36]. In addition, we creatively combined hematopoietic stem cell expansion and endothelial differentiation via the two-step culture system, significantly increasing the yield of EPCs. All the cytokines and growth factors included in the culture system were biologically endogenous factors and clinical-grade reagents that have been proved to carry no risk for cell toxicity or tumor stimulation. Therefore, this ex vivo culture system could provide sufficient autologous EPCs for transplantation treatment in the clinic.

Before entering into clinical trials, new drugs or cell products need a series of proof-of-concept studies, in vivo kinetics, and safety assessment in animal models. Most studies use an immunodeficient animal host to evaluate human cells, but this approach is not very accurate since human cells may behave differently in an animal host environment. Analogous animal models would be more suitable for efficacy and toxicity research. Nonhuman primates should provide a more accurate model for therapeutic evaluation than rodents due to a greater similarity to humans, particularly regarding life-span, hematopoietic system development, and physiological homeostasis. Therefore, we originally conducted this novel autologous transplantation of EPCs in a nonhuman primate model as an in vivo safety and efficacy evaluation. To our knowledge, this is the first report of large-scale generation of primate EPCs ex vivo, achieving an over 7000-fold increase in initial EPCs. In addition, the produced primate EPCs were identified to be similar to human EPCs in terms of the cell morphology and function as assayed by photomicrograph, lectin expression, Dil-Ac-LDL uptake, and NO release. Moreover, based on the CD31/CD144 expression and eNOS/NO levels of the produced cells being dramatically increased after 12 days of culture, it could be possible that EPCs started to differentiate into ECs from day 12; the final produced cells were still a mixture of EPCs and ECs.

Before autologous transplantation in the clinic, it is very helpful to develop a primate model of hepatic sinusoidal endothelium injury. A primate model was constructed with intraperitoneal injection of MCT, a toxic agent that can disrupt the sinusoidal endothelial barrier and stimulate the incorporation of transplanted cells into liver parenchyma. Crucially, endothelial cells play central roles in liver development, organization, repair, and function. Moreover, hepatocytes and liver sinusoidal endothelial cells are the major source of FVIII, which is deficient in hemophilia A. Follenzi and colleagues reported that transplanting healthy liver sinusoidal endothelial cells into hemophilia A mice led to the restoration of plasma factor VIII activity and corrected their bleeding phenotype [8]. If this culture system is combined with ex vivo genome editing technology such as CRISP/Cas-9, sufficient healthy EPCs could be obtained from a small volume of the patient's own mobilized PB. This would minimize the suffering of patients and possibly provide breakthroughs for the complete cure of hemophilia A.

By using the nonhuman primate model to verify the engraftment capacity of generated EPCs/ECs, we applied the FITC-microbeads to label nonhuman primate cells. In our previous testing, these microbeads can tightly bind to the cell surface and prevent fluorescence quenching. The labeling efficiency can reach up to 70% in CD34^+^ cells and 100% in adherent cultured cells. With this unique technique following GFP transfection, nonhuman primate cells were marked by green florescence on the surface as well as in the nucleus. Moreover, to enlarge the signal of the GFP-positive cells, we used an anti-GFP primary antibody followed by a cy3-labeled secondary antibody (red). The double-positive stained cells greatly enhances the assay specificity and identifies engrafted cells, which can be distinguished from cells that have strong autofluorescence such as blood and hepatic cells. When analyzing the double-positive cells on day 7 and day 14 post-transplantation, the results were consistent with a previous study on mice by our group [10], showing that the double-positive cells were present on both day 7 and day 14. However, in the mouse studies, the human EPCs/ECs first adhered onto the vascular inner in the livers of NOD/SCID mice and then gradually migrated to the injured sites. In this present study, the engrafted nonhuman primate cells evenly appeared in the liver tissues from day 7. There could be two explanations: 1) the migration of autologous transplantation in primates is faster than xenotransplantation in mice; and 2) we used more differentiated or matured EPCs/ECs on day 36 of culture, which may result in higher capacities [33, 37] of adherence and more repair of the sites of injury induced by MCT in the hepatic sinusoidal endothelium.

We did not expect to obtain a large number of engrafted cells using this injury model. As we reported, 2–3% of the cells in the monkey liver tissue were transplanted endothelial cells since the transplanted cells (2 × 10^8^) are very limited compared with the total number of hepatic cells. In fact, the hemophilia A patient needs a source that can continually release FVIII rather than for the reconstruction of endothelium. The purpose of this study was to verify that the produced endothelial cells can survive and localize in the injured site without any side effects and that the produced cell preparation ex vivo is feasible for application in the clinical setting. In the future, we plan to perform more functional studies of the produced endothelial cells in a hemophilia A model by directly evaluating their capacity to reverse bleeding. In addition, we will combine FVIII gene correction approaches to develop gene therapies for hemophilia A.

## Conclusion

Large-scale EPCs/ECs were produced from CD34^+^ cells of both human and nonhuman primate peripheral blood ex vivo. Our study strongly indicates that the two-step culture system can be developed into a promising technology platform for industrial production of human and primate EPCs/ECs. The safety and efficacy of this technology in the nonhuman primate model will promote the cell therapy application of ex vivo high-efficient expanded EPCs in ischemic disease and hemophilia A.

## Abbreviations

APC: Allophycocyanin
DAPI: 4,6-Diamidino-2-phenylindole
Dil-ac-LDL: Dil-acetylated low-density lipoprotein
EC: Endothelial cell
eNOS: Endothelial nitric oxide synthase
EPC: Endothelial progenitor cell
FBS: Fetal bovine serum
FITC: Fluorescein isothiocyanate
Flt-3 L: Fms-related tyrosine kinase 3 ligand
G-CSF: Granulocyte-colony stimulating factor
GFP: Green fluorescent protein
Ig: Immunoglobulin
IL: Interleukin
LSEC: Liver sinusoidal endothelial cell
mAb: Monoclonal antibody
MACS: Magnetic-activated cell sorting
MCT: Monocrotaline
MNC: Mononuclear cell
NO: nitric oxide
PB: Peripheral blood
PBS: Phosphate-buffered saline
PE: Phycoerythrin
SCF: Stem cell factor
TPO: Thrombopoietin
VEGF: Vascular endothelial growth factor
